# Online Conversation Monitoring to Understand the Opioid Epidemic: Epidemiological Surveillance Study

**DOI:** 10.2196/17073

**Published:** 2020-06-29

**Authors:** Joshua C Black, Zachary R Margolin, Richard A Olson, Richard C Dart

**Affiliations:** 1 Rocky Mountain Poison and Drug Safety Denver, CO United States

**Keywords:** epidemiological surveillance, infoveillance, infodemiology, opioids, social media, misuse, abuse, addiction, overdose, death

## Abstract

**Background:**

Between 2016 and 2017, the national mortality rate involving opioids continued its escalation; opioid deaths rose from 42,249 to 47,600, bringing the public health crisis to a new height. Considering that 69% of adults in the United States use online social media sites, a resource that builds a more complete understanding of prescription drug misuse and abuse could supplement traditional surveillance instruments. The Food and Drug Administration has identified 5 key risks and consequences of opioid drugs—misuse, abuse, addiction, overdose, and death. Identifying posts that discuss these key risks could lead to novel information that is not typically captured by traditional surveillance systems.

**Objective:**

The goal of this study was to describe the trends of online posts (frequency over time) involving abuse, misuse, addiction, overdose, and death in the United States and to describe the types of websites that host these discussions. Internet posts that mentioned fentanyl, hydrocodone, oxycodone, or oxymorphone were examined.

**Methods:**

Posts that did not refer to personal experiences were removed, after which 3.1 million posts remained. A stratified sample of 61,000 was selected. Unstructured data were classified into 5 key risks by manually coding for key outcomes of misuse, abuse, addiction, overdose, and death. Sampling probabilities of the coded posts were used to estimate the total post volume for each key risk.

**Results:**

Addiction and misuse were the two most commonly discussed key risks for hydrocodone, oxycodone, and oxymorphone. For fentanyl, overdose and death were the most discussed key risks. Fentanyl had the highest estimated number of misuse-, overdose-, and death-related mentions (41,808, 42,659, and 94,169, respectively). Oxycodone had the highest estimated number of abuse- and addiction-related mentions (3548 and 12,679, respectively). The estimated volume of online posts for fentanyl increased by more than 10-fold in late 2017 and 2018. The odds of discussing fentanyl overdose (odds ratios [OR] 4.32, 95% CI 2.43-7.66) and death (OR 5.05, 95% CI 3.10-8.21) were higher for social media, while the odds of discussing fentanyl abuse (OR 0.10, 95% CI 0.04-0.22) and addiction (OR 0.24, 95% CI 0.15-0.38) were higher for blogs and forums.

**Conclusions:**

Of the 5 FDA-defined key risks, fentanyl overdose and death has dominated discussion in recent years, while discussion of oxycodone, hydrocodone, and oxymorphone has decreased. As drug-related deaths continue to increase, an understanding of the motivations, circumstances, and consequences of drug abuse would assist in developing policy responses. Furthermore, content was notably different based on media origin, and studies that exclusively use either social media sites (such as Twitter) or blogs and forums could miss important content. This study sets out sustainable, ongoing methodology for surveilling internet postings regarding these drugs.

## Introduction

Curbing the opioid misuse and abuse epidemic has proven to be a challenging public health problem [1-4]. Although opioids are effective for the management of pain related to acute injury or cancer [5], they have a high potential for causing dependence; opioids are more likely to be abused or misused than other pain management treatments, which has resulted in an alarming increase in the number of overdose deaths compared to the number of other nonprescription opioid-related deaths [5]. The mortality data indicate a continued rise—the national mortality rate involving opioids rose 12.0% from 2016 to 2017 [6]. Oxycodone and hydrocodone are among the most highly dispensed prescription opioids and are also among the most common prescription opioids involved in overdose [7].

The United States Food and Drug Administration (FDA) has responded by identifying 5 key risks and consequences that are part of boxed warnings for opioids: misuse, abuse, addiction, overdose, and death [8]. Understanding the qualitative nature of these risks and consequences (herein termed *key risks*) has also been highlighted as important supportive information to establish context for societal, behavioral, and clinical aspects of risk; this qualitative information could be used to support submissions for abuse-deterrent labeling of opioid products [9]. Reviewing internet postings provides an opportunity to delve into the societal and behavioral causes of the 5 key risks.

One of the more recent additions to public health surveillance of opioids is the monitoring of internet discussions on public blogs, forums, and social media [10]. In contrast to surveys, interviews, or other traditional public health data collection methods, the use of social media, blogs, and forums as a tool for data collection allows for the observation of real-time, unsolicited opinions, feelings, or thoughts [11]. It is possible that online users feel more comfortable sharing covert behaviors in this setting which allows for more truthful perspectives to be shared. Given the danger surrounding some drugs, these unsolicited expressions could shed light in areas that traditional survey instruments cannot. Examples of recent uses of social media data in research include discovering adverse drug events [12], studying addiction [13], tracking the popularity of marijuana concentrates [14], quantifying drug abuse [15], and characterizing discussion surrounding the introduction of an abuse-deterrent product [16].

The Researched Abuse, Diversion and Addiction-Related Surveillance (RADARS) System is a compilation of individual data collection programs that collect product-specific and geography-specific data to form a mosaic understanding of the abuse, misuse, and diversion of prescription drugs [1]. The Web Monitoring Program, established in 2014, focuses on the collection and organization of real-time web content about prescription drugs from over 150 million sites on the internet, including social media, blogs, and forums. The RADARS System Web Monitoring Program combines qualitative and quantitative data collection methods; a team of trained researchers collect daily opioid-related posts and manually code them for variables of interest [16].

The purpose of this study was to characterize the trends of abuse, misuse, addiction, overdose, and death in the United States using internet posts that mention fentanyl, hydrocodone, oxycodone, or oxymorphone and to establish a sustainable ongoing methodology for surveilling internet postings regarding these drugs.

## Methods

### Data Collection

Data were collected by scraping internet posts that mentioned 1 of 4 drugs of interest: fentanyl, hydrocodone, oxycodone, or oxymorphone. These drugs were selected because they have been involved in a variety of behaviors, have been subject to differential regulation, or were frequently prescribed in the United States. Branded oxymorphone and oxycodone products have undergone increasingly restrictive regulation due to findings related to their abuse (such as Opana ER [17] and OxyContin [18]), the potency of fentanyl likely makes it a desirable substance for diversion [19], and hydrocodone is often prescribed within the United States [20]. Posts underwent an algorithmic screening process where posts without substantive content were removed. The remaining posts were sampled and the contents of these posts were reviewed manually and categorized as misuse, abuse, addiction, overdose, or death. Posts that indicate discussion of counterfeit formulations (such as heroin mixed with homemade fentanyl) were excluded prior to analysis.

Data for this project were collected as part of RADARS System ongoing surveillance of the abuse, misuse, and diversion of prescription opioids. All data were collected using a web-crawling platform (Salesforce.com Inc) that scrapes data from public websites that permit content viewing by a third party. Examples of sites that permit this type of crawling include Twitter, Reddit, public blogs and forums, and comment sections on many news sites, while private sites such as personal Facebook pages, Bluelight, and other password-protected sites do not permit this type of crawling. Posts mentioning fentanyl, hydrocodone, oxycodone, or oxymorphone were identified based on specified search-string criteria (such as opioid name, associated misspellings, product names, and unique slang terms) for the 4 opioids (Multimedia Appendix 1). The keywords for each drug substance and product were generated using a phonetic algorithm and then validated using number of hits when entered into a common search engine. Other keywords were identified during the manual coding process.

The study protocol was reviewed and approved by the Colorado Multiple Institutional Review Board prior to the initiation of the RADARS System Web Monitoring Program. Since the publicly available posts were obtained through the Web Monitoring Program and are reported in an aggregated, anonymous manner, it was determined that it was not necessary to consider the Web Monitoring Program as research involving human subjects.

### Data Cleaning

As part of routine web monitoring, posts were screened for predetermined exclusion criteria using a 2-step process. The scraped posts were programmatically screened for predetermined keywords; phrases associated with uninformative posts were excluded. Programmatic exclusions did not remove all uninformative posts; therefore, manual screening for exclusion was also used. The exclusion criteria (for both steps) were defined as posts occurring outside of the surveillance period; in a language other than English; originating outside of the United States; from originating sources other than social media, blogs, or forums; containing the name of a drug of interest that was used in a context unrelated to that drug; that were considered spam (unsolicited online messages); that referenced online pharmacies, news, or pop-culture with no further commentary concerning the drug of interest; or for which the coder was unable to determine a theme. For posts that met one or more exclusion criteria, only the originating posts were removed. If related posts contained informative content (such as a comment mentioning overdose appearing below a news article), then they were not excluded.

Each post was classified by origin as either *social media* or *blogs and forums* based on the originating website. Social media posts originated from sites with a focus on social networking; users on these sites are typically not anonymous, discussions are unguided in nature (ie, not limited to predetermined topics), and commentary is often brief by design (character limits). Examples of social media sites include Twitter, Facebook business pages (which have different privacy rules than those of personal Facebook pages), and Myspace. Blog or forum posts originated from sites that, often, are created to facilitate conversation among users with similar interests; users are often anonymous or not connected to a real-world identity, discussions are topic-specific, and commentary is not limited and can be extensive in nature. Examples of blogs and forums include Reddit, Blogger, and specialized medical forums.

### Sample Design

Due to the very large volume of posts collected, sampling was necessary to identify a subset of posts for manual coding. Posts were required to have occurred between January 1, 2015 and December 31, 2018. A total of 5,048,517 posts were collected for sampling. A stratified random sample without replacement and with proportional allocation was taken from the population of identified posts. Strata included both time (by week) and origin (social media or blogs and forums) of the online posts. If there were less than 2 posts that fell into a given week, that week was folded into a biweekly stratum and weights were adjusted accordingly [21]. The sample size for each opioid was determined based on an expected proportion of 0.05 with a measured precision of 0.015. Sample sizes were selected such that 95% of all confidence intervals of the proportion (calculated from the hypergeometric distribution) obtained the desired precision [22].

### Definitions

A team of 3 trained coders manually reviewed the sample of posts in order to identify reasons for opioid-use outcomes (abuse and misuse), and key medical outcome measures (addiction, overdose, and death). Table 1 contains definitions of the terms as they were used in this study. Extensive training was conducted to ensure that consistent coding was achieved across the team of three coders. A codebook with outlined definitions, examples, and scenarios specific to these data was utilized in the training of each of the 3 coders. Training was complete when the trainee was able to meet the predefined criteria for interrater reliability. These definitions are specific to the RADARS System Web Monitoring Program and may differ from those of other surveillance programs. Each post may contain one or more key risks; are defined as the discussion of any instance of actual misuse, abuse, addiction, overdose, or death involving a drug or drug class of interest; and may include multiple mentions of the opioid or opioids of interest in a single post. Because misuse and abuse were defined similarly, they were coded into a single variable. In any cases of disagreement in coding, the case data were reconciled by an additional coder, and, when necessary, a senior researcher verified the coding.

**Table 1:** Definitions of terms used in the RADARS System Web Monitoring Program.

Term	Definition
Abuse	“A mention that indicates the use of a drug to gain a high, euphoric effect or some other psychotropic effect.”
Addiction	“A mention that indicates one or more of the following: 1) psychological or physical dependence on a drug; 2) tolerance to the psychotropic effects of a drug; 3) withdrawal effects when discontinuing use of a drug.”
Death	“A mention that indicates a death has occurred due to a drug of interest.”
Mention	“Any occasion of a reference to a drug or drug class that appears in a post. One post may contain multiple mentions.”
Misuse	“A mention that indicates the improper or incorrect use of a drug for reason other than the pursuit of a psychotropic effect.”
Overdose	“A mention that indicates the accidental or intentional overdose of a drug, using a dangerous amount of a drug (i.e. a quantity greater than recommended or generally prescribed), or use which may result in a medical intervention.”
Post	“A single point of communication entered by one individual at one specific time point.”

### Statistical Analysis

After manual coding, sampling weights were applied to calculate the estimated number and percentage of posts for each substance in the original population. Odds ratios (OR) and corresponding 95% confidence intervals were calculated from weighted logistic regression for the origin of the posts (social media versus blogs and forums) using all posts in the study period; odds ratios greater than 1.0 indicated higher odds that the post originated from social media. Interrater reliability was calculated between the 3 coders. Predefined acceptability criteria were set, and results were deemed acceptable if 3-way agreement was greater than 90% and if the average coefficient (Gwet AC1) was greater than 0.60 [23]. Statistical analyses were performed in R (3.4.2) and in SAS (version 9.4; SAS Institute Inc).

## Results

### Frequency of Posts Discussing Key Risks

**Figure 1:**
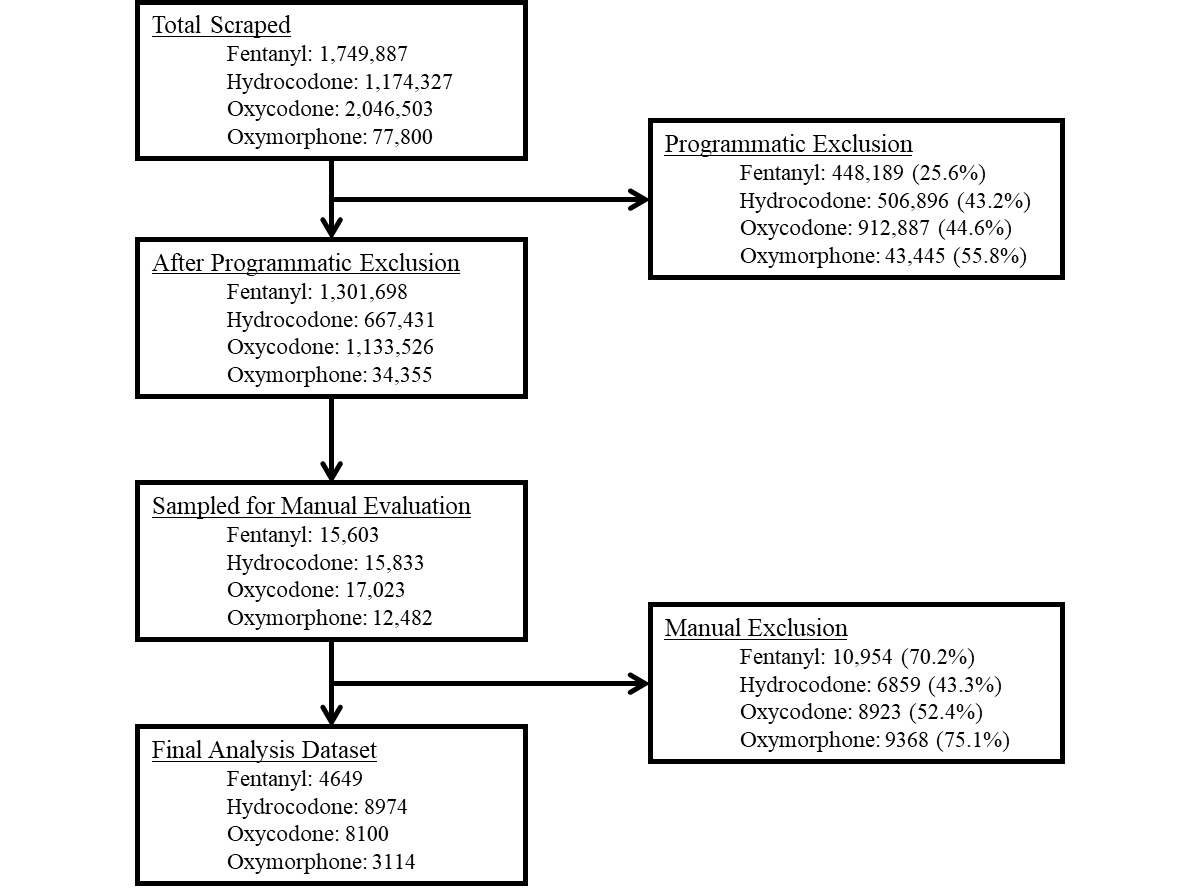
Flowchart of data cleaning process for data collected from the 1st quarter of 2015 through to the 4th quarter of 2018 where percentages represent the proportion of exclusions at each step.

Figure 1 depicts the data cleaning process and provides the number of opioid-specific mentions that were sampled and the number of mentions that were analyzed after exclusion criteria were applied. In the final sample (n=24,837), the outcomes were infrequently observed. Out of all posts for all drugs, 1.95% (485/24,837) mentioned misuse, 0.67% (166/24,837) mentioned abuse, 2.35% (584/24,837) mentioned addiction, 1.53% (379/24,837) mentioned overdose, and 2.15% (534/24,837) mentioned death.

Interrater reliability was acceptable according to both criteria for all the coding variables. Three-way percent agreement was high for misuse and abuse (97.8%), addiction (99.4%), overdose (99.9%), and death (99.6%). Gwet AC1 coefficient was also high for misuse and abuse (0.98), addiction (0.99), overdose (1.0), and death (1.0)

**Figure 2:**
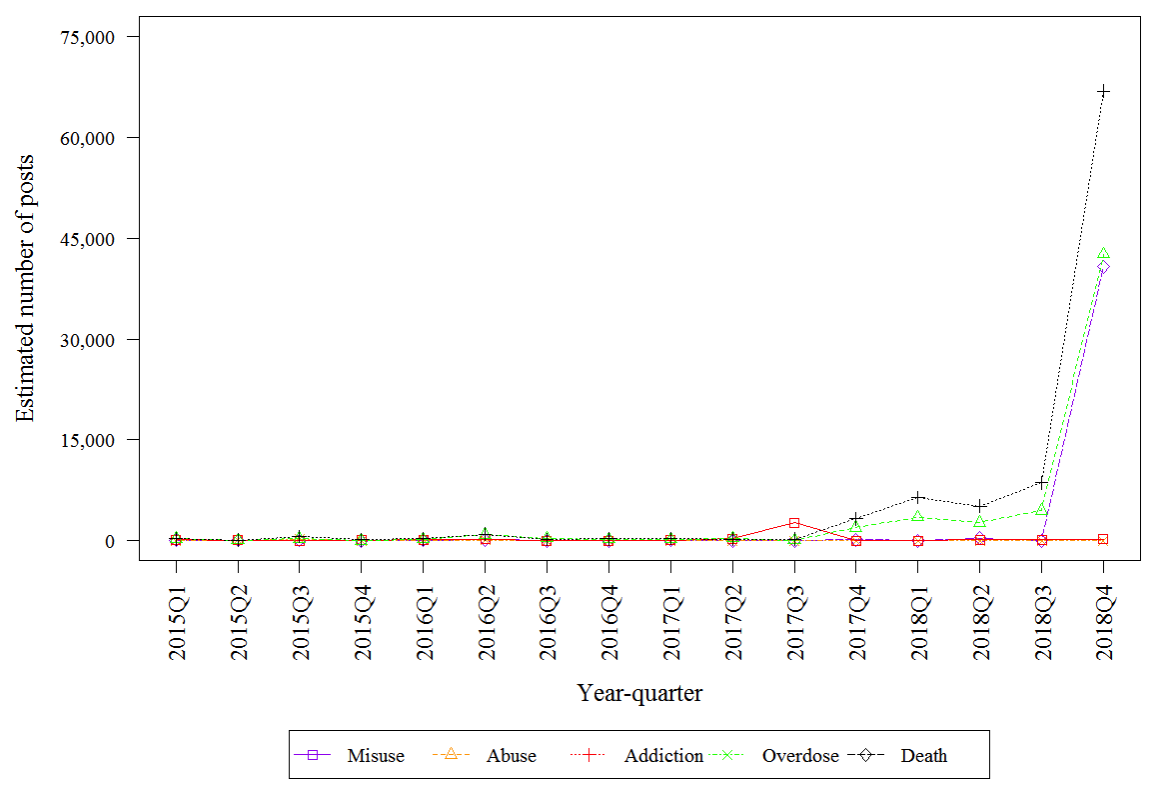
Estimated number of quarterly fentanyl posts.

**Figure 3:**
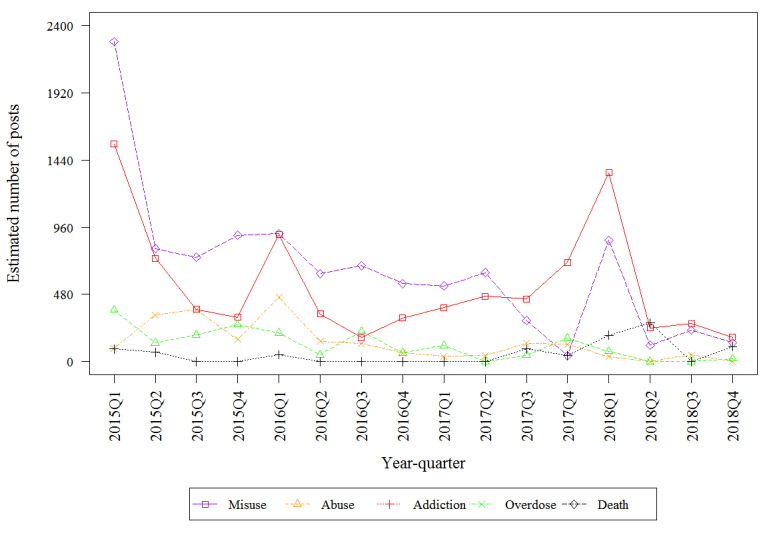
Estimated number of quarterly hydrocodone posts.

**Figure 4:**
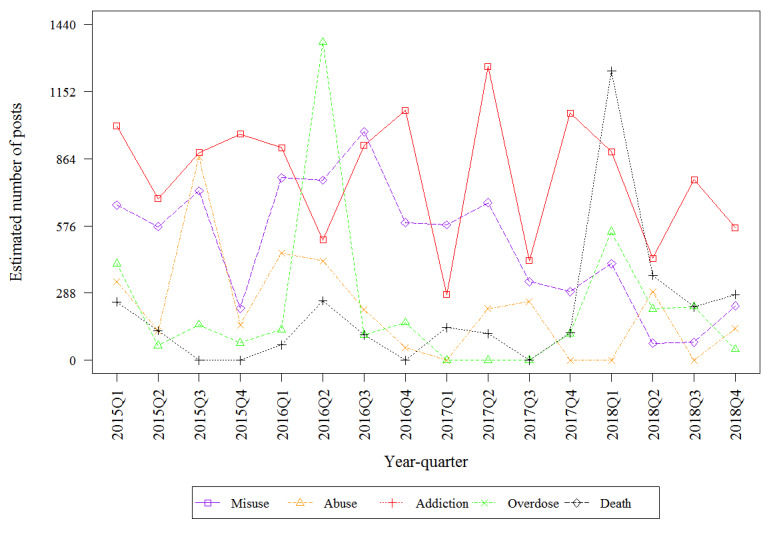
Estimated number of quarterly oxycodone posts.

**Figure 5:**
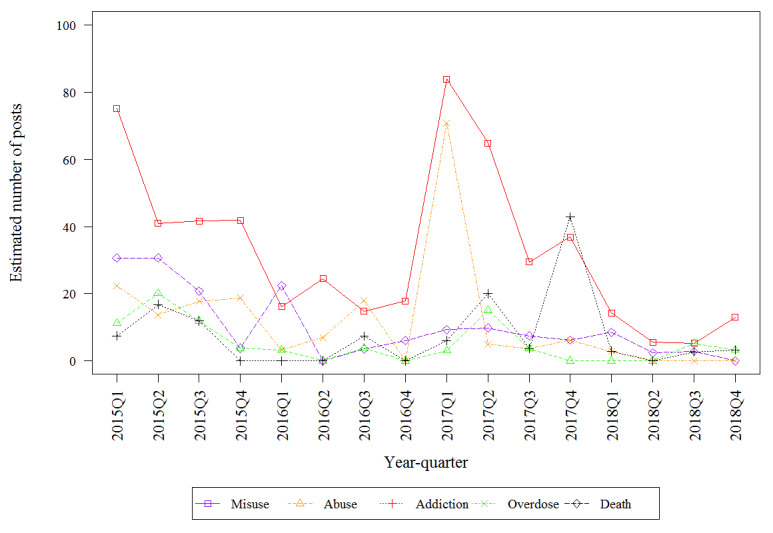
Estimated number of quarterly oxymorphone posts.

Table 2 describes the estimated number of mentions by drug on the public internet that discuss the 5 key risks. The top 3 highest estimated frequencies involved fentanyl (death, overdose, and misuse). Misuse had the highest estimated frequency for hydrocodone. Addiction had the highest estimated frequency for oxycodone. All key risks involving oxymorphone were infrequently discussed, with fewer than 600 posts discussing each of the 5 key risks. Fentanyl-related death had the highest estimated frequency of any key risk–drug combinations which was from 10- to 100-fold higher than the frequency of death mentions for other drugs. Figure 2, Figure 3, Figure 4, and Figure 5 describe the estimated number of posts (per 10,000 posts) for fentanyl, hydrocodone, oxycodone, and oxymorphone, respectively, throughout the surveillance period. The estimated number of posts discussing misuse, abuse, and addiction generally decreased across the study period for hydrocodone, oxycodone, and oxymorphone. Discussions of fentanyl misuse, overdose, and death surged at the end of 2017 and continued to surge through 2018.

**Table 2:** Number of posts analyzed in samples and corresponding population estimates.

Key risks	Drug Mentions										
	Fentanyl		Hydrocodone				Oxycodone			Oxymorphone	
	Sample analyzed^a^ (n=4649), n	Population estimate^b^, n (95% CI)		Sample analyzed, n (n=8974)	Population estimate, n (95% CI)	Sample analyzed, n (n=8100)		Population estimate, (95% CI)	Sample analyzed, n (n=3114)		Population estimate, n (95% CI)
Abuse	24	627 (351-902)		42	2181 (1503-2858)	43		3548 (2424-4672)	57		189 (140-239)
Misuse	130	41808 (34,058-49,559)		199	10379 (8857-11,900)	107		7997 (6393-9601)	49		165 (118-211)
Addiction	73	4435 (3209-5662)		183	8766 (7419-10,113)	176		12679 (10,721-14,637)	152		526 (442-610)
Overdose	271	42659 (34,750-50,568)		36	1911 (1257-2564)	48		3633 (2583-4682)	24		84 (50-119)
Death	427	94169 (83,575-104,763)		23	913 (514-1312)	47		3291 (2326-4256)	37		125 (84-166)

^a^Sample analyzed refers to the number of posts manually reviewed by the team of coders.

^b^Population estimate refers to the extrapolated number of posts.

### Origin of Posts Discussing Key Risks

**Figure 6:**
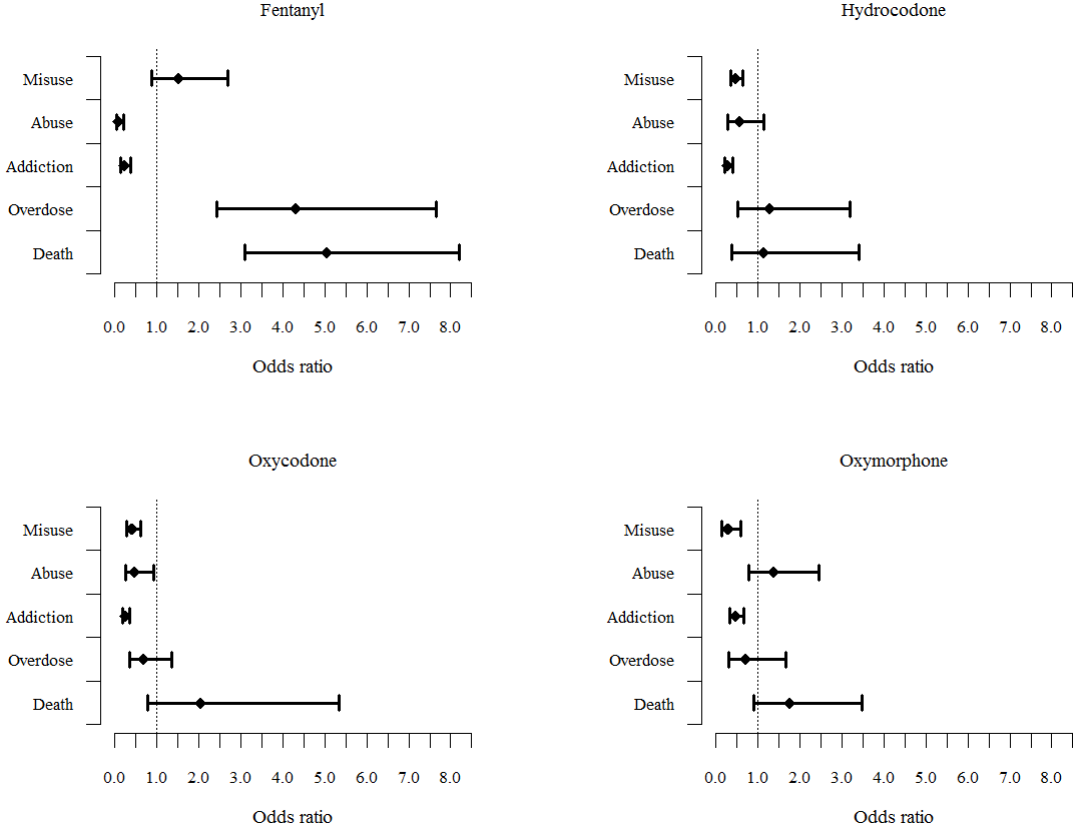
Post origin by key risk across drugs. Odds ratios greater than 1.0 indicate higher likelihood of discussion on social media.

Substantial proportions of posts discussing key risks originated outside of social media. An estimated 48.0% (95% CI 46.2%-49.8%) of oxymorphone posts, 16.8% (95% CI 15.9%-17.6%) of oxycodone posts, 18.3% (95% CI 17.5%-19.1%) of hydrocodone posts, and 14.6% (95% CI 13.6%-15.6%) of fentanyl posts originated from blogs and forums. Out of all social media posts discussing key risks, Twitter was a substantial origin. An estimated 19.9% (95% CI 18.9%-20.8%) of oxymorphone social media posts, 49.8% (95% CI 48.9%-50.7%) of oxycodone social media posts, 62.8% (95% CI 61.9%-63.7%) of hydrocodone social media posts, and 30.6% (95% CI 29.8%-31.4%) of fentanyl social media posts originated from Twitter. Odds ratios for the origin of discussion of key risks are shown in Figure 6. For ease of presentation, all odds ratios refer to social media as the reference. For all drugs, the estimated odds of discussing addiction were higher in blogs and forums than in social media. Odds of posting about addiction on social media were smaller than on blogs and forums; odds ratios ranged from (fentanyl) 0.24 (95% CI 0.15-0.38) to (oxymorphone) 0.46 (95% CI 0.32-0.67). Conversely, the estimated odds of discussing death were higher in social media than in blogs and forums for all drugs. The odds ratios for discussions of misuse, abuse, and overdose also differed by drug. Notably there was a distinct separation of fentanyl abuse and addiction discussions from fentanyl overdose and death discussions. Odds of discussing fentanyl overdose and death were higher for social media (overdose: OR 4.32, 95% CI 2.43-7.66; death OR 5.05, 95% CI 3.10-8.21), while odds of discussing fentanyl abuse and addiction were higher for blogs and forums (abuse: OR 0.10, 95% CI 0.04-0.22; addiction: OR 0.24, 95% CI 0.15-0.38). For other drugs, odds of discussing misuse were higher in blogs and forums.

## Discussion

### Principal Findings

The purpose of this study was to demonstrate a sustainable, ongoing methodology of evaluating internet posts that mention drugs by presenting the trends of online discussion regarding abuse, misuse, addiction, overdose, and death and documenting the post origin of these key risks. The estimated numbers of posts that discussed misuse, abuse, and addiction decreased for hydrocodone, oxycodone, and oxymorphone over the surveillance period, which is not surprising since prescription guidelines have tightened the availability of these drugs [24]. Discussion of fentanyl rose sharply in 2017 and 2018, coincident with increased fentanyl mortality, increased illicit fentanyl production, and public awareness campaigns about the dangers of fentanyl. Further content evaluation of fentanyl posts could elucidate specific societal events that caused the sudden increase. Three strengths of the study results presented herein that distinguished this surveillance system from other opioid abuse internet surveillance programs [10] were that content coverage included originating sources beyond social media; the focus was on the FDA key risks—misuse, abuse, addiction, overdose, and death; and the originating sources of these key risk discussions were identified. It was noted that discussions of several key risks were more likely to be found on blogs and forums than on social media. This could result in selection bias in studies that focus only on social media. For example, if a study on substance-use behavior involving these four opioids used only social media, the post content would select against discussions of addiction and addiction-related behaviors.

Anonymity has a profound influence on the likelihood of revealing sensitive behaviors [25]. Traditional public health surveillance programs typically rely on confidentiality, and use of anonymous posts and social media may allow for reporting of behaviors or opinions not normally captured by traditional systems. Moreover, the diversity of online data sources used in this study allowed more comprehensive assessment of online discussion content than that which would have been allowed with an approach that targeted a single social media site (such as Twitter) or forum (such as drugs-forum). One review of studies that collected data on illicit drug use from a variety of platforms [10] found 14 studies that met inclusion criteria. Using our definition of media originating source, only 2 of those studies collected data from blogs and forums. Our results show that the discussions on social media often involve different information or outcomes compared to those on blogs and forums. There could be several reasons for this; social media posts were more likely to discuss overdose and death, outcomes that could elicit strong emotion and vocal responses. Blogs and forums were more related to misuse, abuse, and addiction, outcomes that could generate less strong response. We speculate the blogs and forums tend to have larger character limits, and could be better suited for complex topics, such as addiction. As social media are commonly associated with an actual identity of the user, it is frequently less anonymous and can easily be searched by employers, family, and friends; this might drive discussion of stigmatizing behaviors, such as misuse, abuse, and addition, to blogs and forums where individuals can use pseudonyms. Research focused on overdose and death outcomes will likely find more valuable data on social media, while research around misuse, abuse, and addiction should look toward blogs and forums; however, a mosaic approach should ideally include both originating sources. Our results indicated that content originated from different sectors of the internet for addiction and death discussions, and qualitative analyses that focus only on subregions of the internet could miss important information on these key risks.

Due to the unsolicited nature of internet postings, qualitative analysis of web content can be used to identify unknown knowledge gaps in substance-abuse research. For future research efforts using the method reported here, data can be examined for polysubstance use, methods of tampering with abuse-deterrent formulations, or low-frequency side effects. Furthermore, negative outcomes are not the only topic to study. Events can stimulate online discussion that supports the proper therapeutic use of a drug or discussions can compare the efficacy of similar drug products.

### Limitations

One limitation of this study is that only publicly available websites were studied. Unique information likely exists on websites with policies that prevent public scraping (such as most of Facebook or Bluelight). The second limitation was the unstructured nature of the raw data and the potential ambiguity associated with manually coding these key risks, which was addressed by team meetings to ascertain group consensus. Furthermore, interrater reliability was assessed and found to be satisfactory. Finally, separating illicit from licit fentanyl was challenging, and likely much of the discussion referred to illicit instead of licit fentanyl.

### Conclusions

Use of internet posts reveals a unique perspective to the opioid epidemic that is not found using traditional surveillance systems and can be a gateway to understanding qualitative aspects of drug use. Anonymity and the unsolicited nature of these data offer advantages to understanding emerging trends. Surveillance of diverse content providers should be used to understand how policy or other interventions are received by the broader community.

## Appendix

Multimedia Appendix 1 Search keyword examples.

## Abbreviations

FDA: Food and Drug Administration
RADARS: Researched Abuse, Diversion and Addiction‐Related Surveillance
