# Supplementary material for: Online Conversation Monitoring to Understand the Opioid Epidemic: Epidemiological Surveillance Study
Source: JMIR Public Health Surveill. 2020 Jun 29;6(2):e17073. doi: 10.2196/17073 (PMC7367521; doi:10.2196/17073)
Supplement: Multimedia Appendix 1 [file publichealth_v6i2e17073_app1.docx]

**Table 1: Example opioid active ingredients and corresponding keywords.**

| Drug | Example Keywords |
| --- | --- |
| Fentanyl | fentanyl, fintanil, fintenyl, fentinyl, fentenyl, fentenil, fentanyls, fantanyl |
| Hydrocodone | hydrocodone, hidrocodone, hidrocodon, hydracodone, hydracodon, hydrocodon, hydrocodones |
| Oxycodone | oxycodone, oxicodone, oxycodon, oxykodone, oxycodones, oxycoden |
| Oxymorphone | oxymorphone, oxymorphine, oxymorfone, oxymorphene, oxymorophone, oxymorphones |

*All available branded products containing each active ingredient in the United States market are included as keywords.*
